# The magnitude, but not the duration of elevated central venous pressure is associated with mortality in sepsis patients: An analysis of the MIMIC-IV database

**DOI:** 10.1371/journal.pone.0281549

**Published:** 2023-02-08

**Authors:** Han Chen, Shu-Rong Gong, Xiu-Ling Shang, Jun Li, Rong-Guo Yu

**Affiliations:** Department of Critical Care Medicine, Fujian Shengli Clinical Medical College of Fujian Medical University, Fujian Provincial Key Laboratory of Critical Care Medicine, Fujian Provincial Hospital, Fuzhou, Fujian, China; Sohag University Faculty of Medicine, EGYPT

## Abstract

**Background:**

It is unclear whether the magnitude and duration of elevated central venous pressure (ECVP) greater than ten mmHg has the same impact on mortality in sepsis patients.

**Methods:**

Critically ill patients with sepsis were identified from the Medical Information Mart for Intensive Care (MIMIC)-IV database. The duration and the magnitude of ECVP were calculated. Normalized ECVP load was defined as the ECVP load (the sum of ECVP value times its duration) divided by the total duration of ECVP. The primary endpoint was 28-day mortality. Kaplan-Meier survival analysis was used to compare survival between patients with high or low normalized ECVP load.

**Results:**

A total of 1071 sepsis patients were included. Higher normalized ECVP load was associated with higher mortality rate; in contrast, the duration of ECVP was not associated with mortality. A linear relationship between normalized ECVP load and mortality was identified. Patients with higher normalized ECVP load had less urine output and more positive fluid balance.

**Conclusion:**

The magnitude, but not the duration of ECVP, is associated with mortality in sepsis patients. ECVP should be considered as a valuable and easily accessible safety parameter during fluid resuscitation.

## Introduction

Sepsis and septic shock remain the main causes of admission to the intensive care unit (ICU) and death in critically ill patients [1]. During the first phase of treatment ("the salvage phase" [2]), one of the main therapies is fluid administration to restore blood pressure and cardiac output. Intravenous infusing at least 30 mL/kg of crystalloids within the first three hours of resuscitation is recommended by the latest updated Surviving Sepsis Campaign (SSC) guidelines [3]. Criticisms have been raised since a fixed, large volume of fluid infusion could result in fluid overload [4].

Central venous pressure (CVP) is often misinterpreted as an indicator of intravascular volume status. From the (patho)physiological point of view, CVP is determined by the interaction of cardiac function and venous return function, as suggested by Guyton et al. [5], and therefore does not merely reflect the intravascular volume status. Small changes in CVP along with increases in cardiac output indicate fluid responsiveness, which would thus lead to fluid administration in sepsis patients with evidence of tissue hypoperfusion. In contrast, significant increases in CVP with little changes in cardiac output suggest poor tolerance to fluids, in which case patients are at high risk of fluid overload and may benefit little or even harmed from fluids.

It has been shown by numerous studies that increased CVP level is associated with increased risk of mortality [6–9] and acute renal failure (AKI) [6, 10, 11]. However, previous studies focused mainly on the magnitude of the elevation of CVP, but the effect of the duration of such elevation was less appreciated. One retrospective study of a heterogeneous ICU population showed that the duration of elevated central venous pressure (ECVP) was higher in the non-survivors than in the survivors [7]. Indeed, a greater intensity of exposure to ECVP must be affected by either increase in the magnitude or extension of the duration of ECVP, or both. We evaluated the effect of the magnitude and duration of ECVP on mortality in sepsis patients by analyzing the data from a large, public, de-identified clinical database.

## Methods

### Database and ethics

The data of the present study were collected from the Medical Information Mart for Intensive Care IV (MIMIC IV) database [12]. MIMIC-IV was published on March 16^th^, 2021, an update to the MIMIC-III database [13]. It contains de-identified data of patients who stayed in critical care units of the Beth Israel Deaconess Medical Center between 2008 and 2019. The establishment of the database was approved by the institutional review boards of the Massachusetts Institute of Technology (Cambridge, MA) and Beth Israel Deaconess Medical Center (Boston, MA). Consent was obtained for the original data collection. Therefore, the Institutional Review Board of Fujian Provincial Hospital waived the informed consent for the present study. Data were extracted by Dr. Han Chen and Dr. Shu-Rong Gong on May 1^st^, 2021 (database access certification number: HC 36014736, SRG 35606844). The study was designed and conducted in accordance with relevant guidelines and regulations (Declaration of Helsinki).

### Data extraction

PostgreSQL tools V.10.16 was used for data extraction as previously reported [14, 15]. The following data were extracted by using Structured Query Language (SQL): age, gender, weight, co-morbidities, the survival time, length of hospital stay, and length of ICU stay, sequential organ failure assessment (SOFA) score, simplified acute physiology score-II (SAPS-II), vital signs, first-day laboratory results, daily fluid input, fluid balance, urine output, and the present of AKI and septic shock. Besides, the time and value of CVP measurement in the ICU were also extracted for further calculation.

### Population and exposure

All patients with sepsis were screened for inclusion. The inclusion criteria were: 1) Patients fulfilled the definition of septic shock according to the sepsis-3.0 criteria [1]; 2) Age ≥ 18 years; 3) CVP data available. The exclusion criteria were: 1) CVP measurement started after 24 hours of ICU admission; 2) CVP measurement ended before 72 hours of ICU stay.

**Fig 1:**
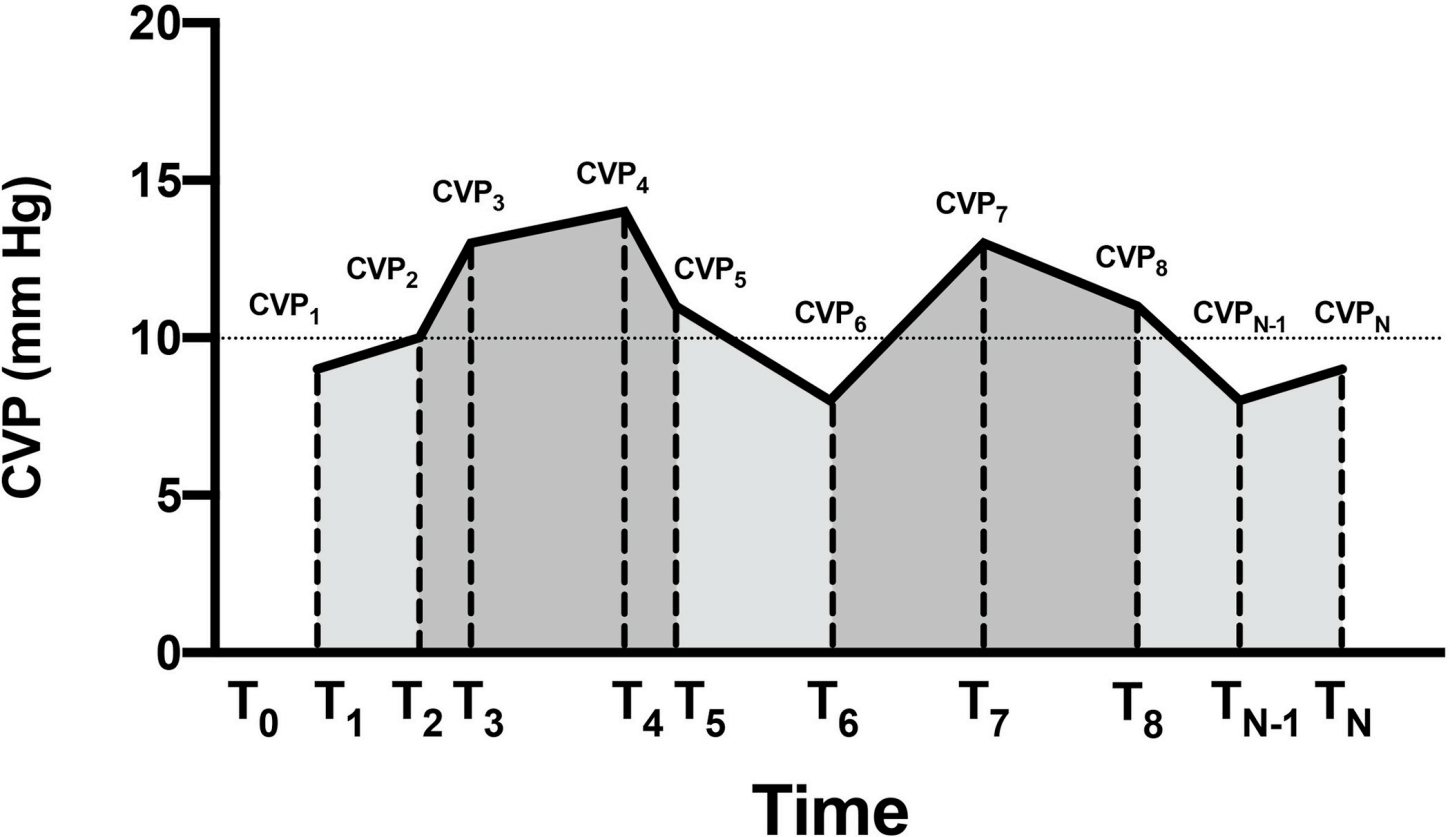
Diagram of the calculation of central venous pressure loads. T_0_ is the time of ICU admission, and T_N_ is the time of the last CVP measurement within the first 72 hours of ICU stay. Central venous pressure (CVP) load is calculated as the area under the CVP curve, which equals to (CVP_2_ + CVP_1_)/2 × (T_2_ –T_1_) + (CVP_3_ + CVP_2_)/2 × (T_3_ –T_2_) +…+ (CVP_N_ + CVP_N-1_)/2 × (T_N_−T_N-1_). CVP duration is the total time of CVP monitoring, which equals to (T_N_−T_1_). Normalized CVP load is thus CVP load divided by CVP duration. Elevated CVP (ECVP) is defined as the average CVP level of two adjacent time points > 10 mmHg, and the ECVP load is defined as the sum of ECVP areas (areas in dark gray). ECVP duration is the sum of the corresponding time interval of ECVP (i.e., (T_2_ –T_1_) + (T_6_ –T_5_) + (T_N_−T_8_) in the diagram). Normalized ECVP load is thus ECVP load divided by ECVP duration.

Only the CVP data before 72 hours of the ICU stay were analyzed. We calculated the following parameters using the trapezoidal rule: CVP duration, CVP load, normalized CVP load, ECVP duration, ECVP load, and normalized ECVP load. In brief, a “load” is the area under the CVP curve, reflecting the overall exposure to CVP/ECVP. A “normalized load” is thus the area divided by the corresponding period (see Fig 1 for a detailed explanation). ECVP was defined as CVP above the level of 10 mmHg, which is a relatively higher CVP level that was considered potentially deleterious [7, 16]. Averaged ECVP load during CVP monitoring was calculated as ECVP load divided by CVP duration. The primary outcome was 28-day mortality.

### Statistical analysis

Kolmogorov-Smirnov test was used for the assessment of the normality of distribution. Continuous variables were presented as mean with standard deviation for normal distribution or median with interquartile ranges (IQR) for normal distribution. Student’s *t*-test or Wilcoxon rank-sum test were used as appropriate. Categorical variables were presented as counts (percentages) and compared using the chi-square test. We used a locally weighted smoothing (Lowess Smoothing) technique to explore the crude relationship between normalized CVP/ECVP and mortality. We divided the overall population into the high and low normalized ECVP load groups base on the median of normalized ECVP load. Kaplan-Meier survival analysis was performed and tested by the Log-Rank test. Multivariate logistic regression analysis was performed using foreword procedures with factors showing *p* < 0.20 in univariate analysis. STATA (ver. 15.1, StataCorp., TX, USA) was used for data analysis. All reported *p-*values were two-sided, and a *p* < 0.05 was considered significant.

## Results

**Fig 2:**
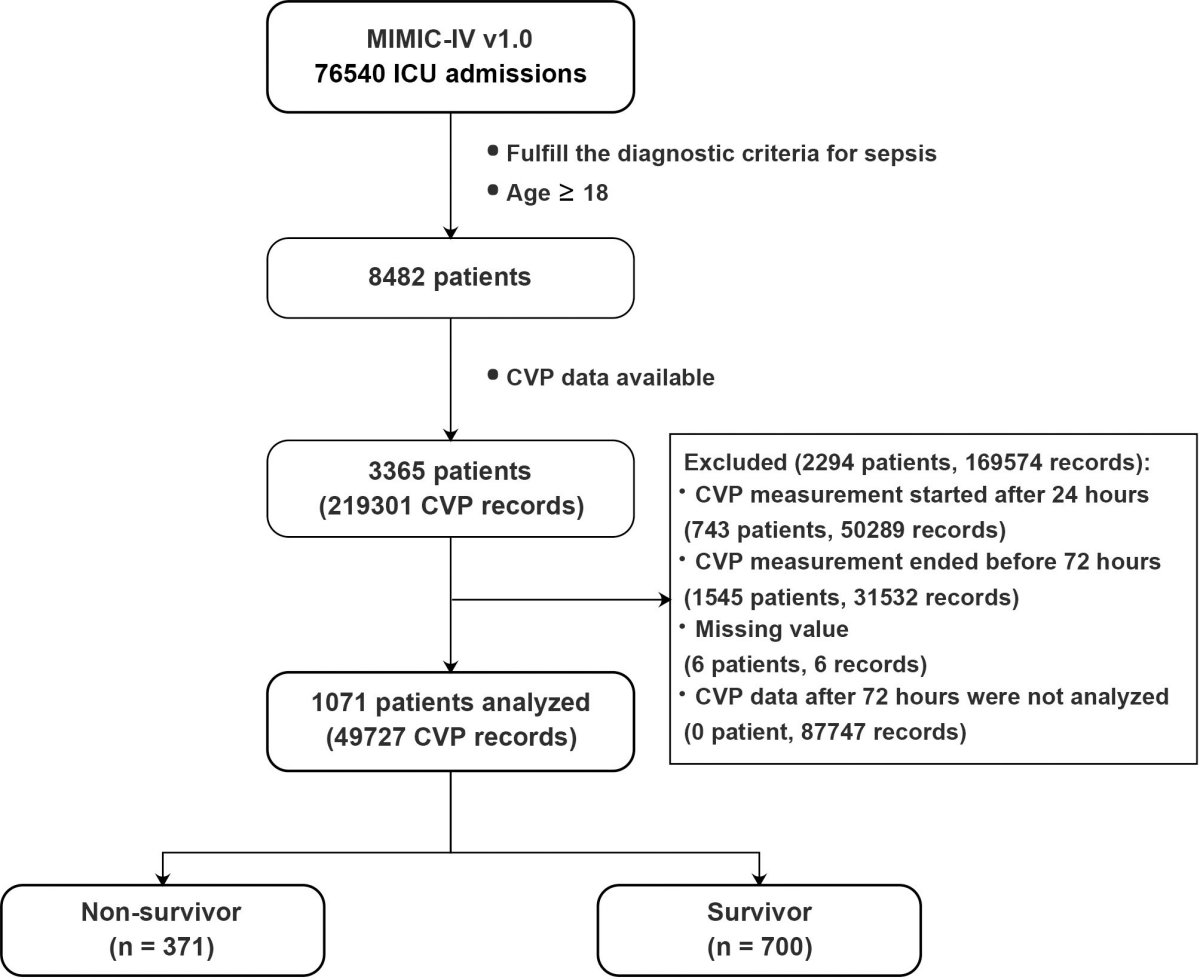
Flowchart showing a step-by-step selection of patients included in the study. *CVP* central venous pressure, *ICU* intensive care unit.

A total of 1071 patients were included, and the 28-day mortality was 39.8% (700 survivors, 371 non-survivors, Fig 2). Baseline patient characteristics are summarized in Table 1. In brief, non-survivors were older (69 ± 13.7 *vs*. 64.4 ± 15.9 years old, *p* < 0.001), and had higher SAPS-II and SOFA score (55.6 ± 13.2 *vs*. 49.4 ± 14.4, and 9.8 ± 3.5 vs. 8.7 ± 3.5, respectively; all *p* < 0.001). Non-survivors were more likely to have underlying diseases, including congestive heart failure, liver disease, renal disease, diabetes and malignant tumors (Table 1). Detailed baseline characteristics are presented in Table E1 in S1 File.

**Table 1:** Comparisons of the baseline clinical characteristics between survivors and non-survivors in the first 24 hours.

	Survivor (n = 700)	Non-survivor (n = 371)	Overall population (n = 1071)	*p* value
Age (year)	64.4 ± 15.9	69 ± 13.7	66 ± 15.3	< 0.001
Female	283 (40.4)	167 (45)	450 (42)	0.148
Weight (kg)	84.1 ± 24.6	85.7 ± 43.3	84.7 ± 32.3	0.445
SOFA score	8.7 ± 3.5	9.8 ± 3.5	9.1 ± 3.6	< 0.001
SAPS-II score	49.4 ± 14.4	55.6 ± 13.2	51.6 ± 14.3	< 0.001
**Comorbidities**				
Congestive heart failure	284 (40.6)	177 (47.7)	461 (43)	0.025
Cerebrovascular disease	72 (10.3)	38 (10.2)	110 (10.3)	0.982
Chronic pulmonary disease	207 (29.6)	131 (35.3)	338 (31.6)	0.055
Mild liver disease	167 (23.9)	112 (30.2)	279 (26.1)	0.025
Severe liver disease	62 (8.9)	54 (14.6)	116 (10.8)	0.004
Renal disease	186 (26.6)	129 (34.8)	315 (29.4)	0.005
Diabetes without complication	187 (26.7)	122 (32.9)	309 (28.9)	0.034
Diabetes with complication	63 (9)	37 (10)	100 (9.3)	0.603
Malignant cancer	71 (10.1)	56 (15.1)	127 (11.9)	0.017
AIDS	9 (1.3)	2 (0.5)	11 (1)	0.249
**Laboratory results in the first 24 hours**				
Maximum lactate (mmol/L)	3.8 ± 2.9	4.6 ± 3.5	4.1 ± 3.2	< 0.001
Minimum albumin (g/dL)	2.6 ± 0.7	2.7 ± 0.7	2.6 ± 0.7	0.285
Maximum bilirubin (mg/L)	2.4 ± 3.8	3.3 ± 5.8	2.7 ± 4.7	0.011
Maximum creatinine (mg/dL)	2.1 ± 1.6	2.4 ± 1.7	2.2 ± 1.6	0.028
Maximum white blood cell count (K/uL)	18.3 ± 11.7	18.9 ± 14.2	18.5 ± 12.6	0.451
Minimum hemoglobin (g/dL)	9.4 ± 2.1	9.1 ± 2	9.3 ± 2.1	0.008
Minimum platelet (K/uL)	193.6 ± 138.9	167.3 ± 116.9	184.5 ± 132.2	0.002
Maximum activated partial thromboplastin time (sec)	55.8 ± 35.4	58.7 ± 36.3	56.8 ± 35.7	0.212
Maximum international normalized ratio	1.9 ± 1.2	2.3 ± 1.7	2.1 ± 1.4	< 0.001
**Vital signs**				
Mean heart rate (bpm)	94.4 ± 17.7	94.8 ± 17.3	94.5 ± 17.5	0.697
Minimum mean arterial blood pressure (mmHg)	49 ± 14.4	46.4 ± 14.4	48.1 ± 14.4	0.006
Mean respiratory rate (bpm)	21.5 ± 4.5	21.5 ± 4.3	21.5 ± 4.4	0.947
Mean body temperature (°C)	37 ± 0.8	36.8 ± 0.8	36.9 ± 0.8	0.001
Minimum pulse O_2_ saturation (%)	90.2 ± 9.3	88.6 ± 9.5	89.6 ± 9.4	0.008

Data are presented as mean ± standard deviation or median (interquartile range) for continuous variables and counts (percentages) for categorical variables.

*AIDS* acquired immunodeficiency syndrome, *SAPS-II* simplified acute physiology score-II, *SOFA* sequential organ failure assessment.

**Fig 3:**
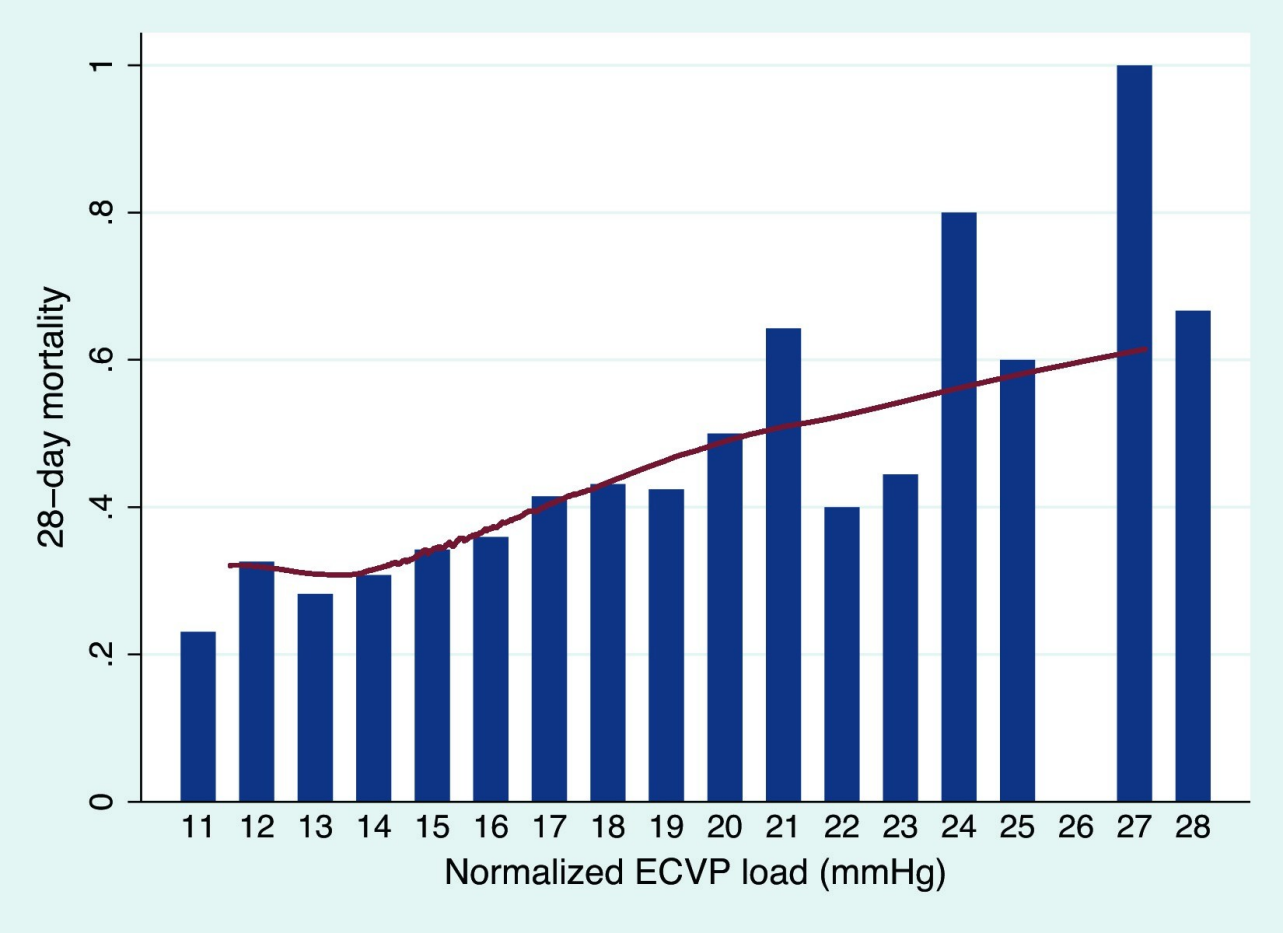
Mortality rate in patients with different normalized elevated central venous pressure load. The bar plot demonstrates the raw mortality rate in each patient subgroup with different normalized elevated central venous pressure (ECVP) load. Locally weighted smoothing (Lowess Smoothing) showed a linear relationship between normalized ECVP load and mortality rate. Note that the number of patients with a normalized ECVP load equals 26 mmHg was zero.

Survivors had a longer duration of CVP monitoring than non-survivors. Normalized CVP load and normalized ECVP load were significantly lower in survivors than in non-survivors. The averaged ECVP load during the entire period of CVP monitoring was also significantly lower in survivors than in non-survivors. There was no significant difference in the duration of ECVP between survivors and non-survivors, and there was also no significant difference in the proportion of ECVP duration to CVP duration. CVP load and ECVP load were also comparable (Table 2). A linear relationship between normalized ECVP load and the mortality rate was observed when Lowess Smoothing was performed (Fig 3). Similarly, a positive correlation between normalized CVP load and mortality was also found (Figure E1 in S1 File).

**Table 2:** Comparisons of the duration and the load of central venous pressure between survivors and non-survivors.

	Survivor (n = 700)	Non-survivor (n = 371)	Overall population (n = 1071)	p value
CVP duration (hour)	61.4 ± 11.1	58.9 ± 13.6	60.6 ± 12.1	0.001
ECVP duration (hour)	37.7 ± 20.5	38.2 ± 20.8	37.9 ± 20.6	0.692
Percentage of ECVP duration in total CVP duration (%)	61.1 ± 31.2	64.9 ± 32.1	62.4 ± 31.6	0.055
CVP load (mmHg·hr)	757.9 ± 287.7	771.6 ± 327.3	762.6 ± 301.9	0.480
ECVP load (mmHg·hr)	569.1 (278.3, 865)	639.3 (287.1, 909)	589 (280, 882)	0.153
Normalized CVP load (mmHg)	12.3 ± 4	13.1 ± 4.6	12.6 ± 4.3	0.002
Normalized ECVP load (mmHg)	14.1 (12.9, 15.9)	14.8 (13.1, 17.1)	14.3 (13, 16.2)	<0.001
Averaged ECVP load during CVP monitoring (mmHg)	9.2 (4.6, 13.9)	11.1 (5.5, 15.6)	9.8 (4.8, 14.4)	<0.001

Data are presented as mean ± standard deviation or median (interquartile range).

*CVP* central venous pressure; *ECVP* elevated central venous pressure.

**Fig 4:**
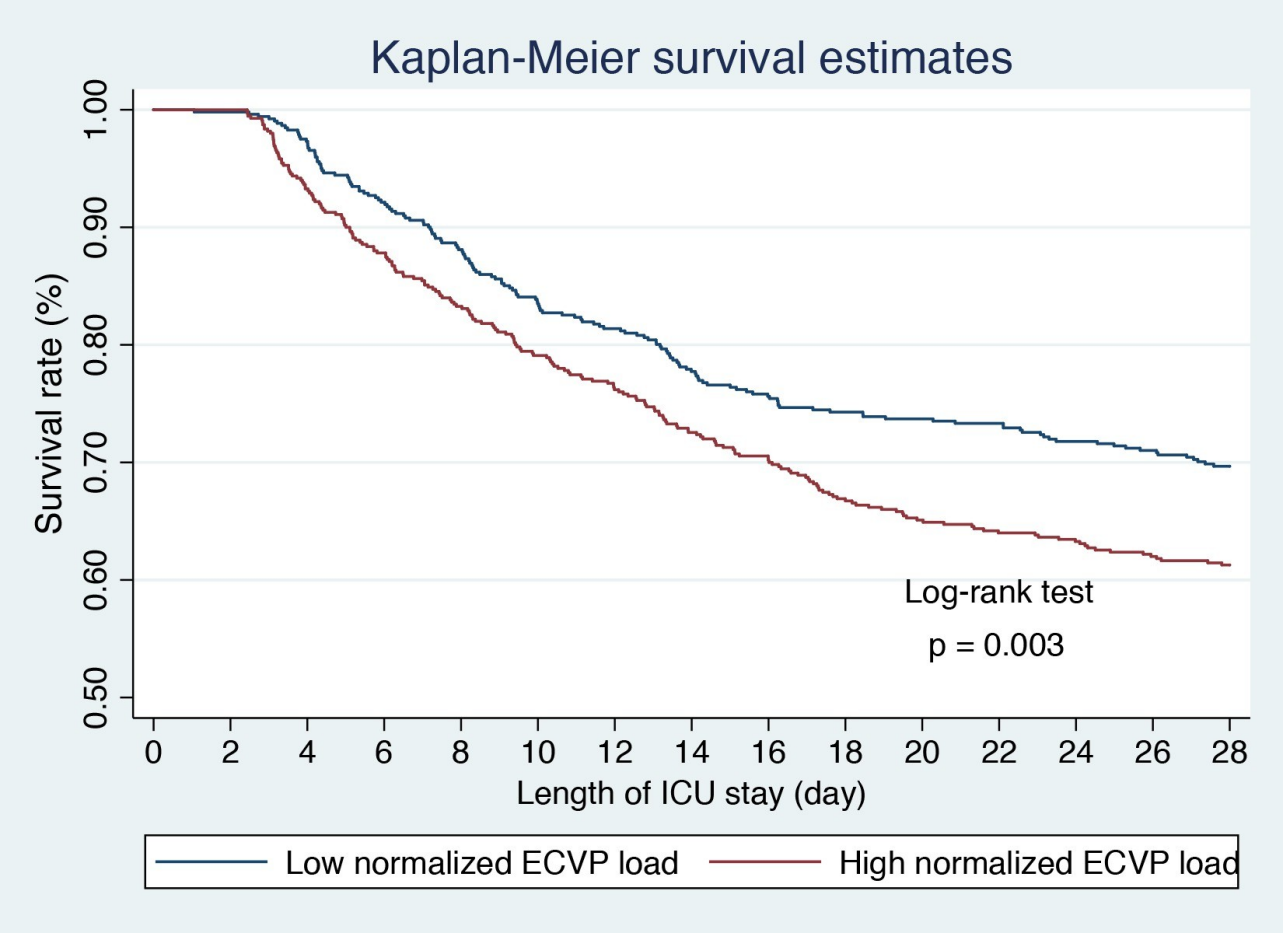
Kaplan-Meier survival analysis of patients with high or low normalized elevated central venous pressure load. Patients were divided into the high normalized elevated central venous pressure (ECVP) load group (n = 550) and the low normalized ECVP load group (n = 521) according to the median of normalized ECVP load. The 28-day mortality rate was significantly higher in patients with higher normalized ECVP loads than those with lower normalized ECVP loads.

**Fig 5:**
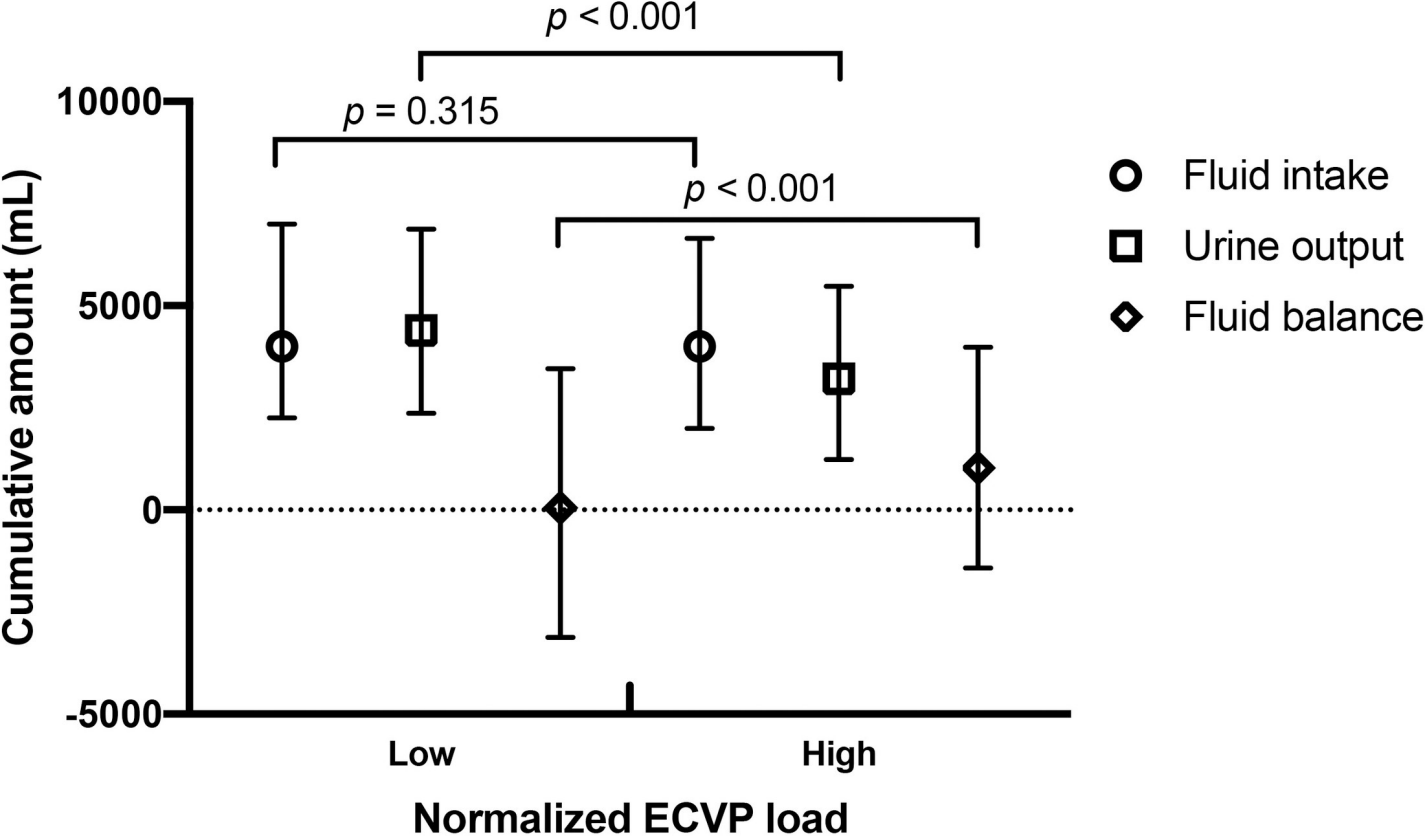
Fluid balance of patients with high or low normalized elevated central venous pressure load. Patients were divided into the high normalized elevated central venous pressure (ECVP) load group (n = 550) and the low normalized ECVP load group (n = 521) according to the median of normalized ECVP load. Data were presented as median and interquartile range. The high normalized ECVP load group had significantly less cumulative urine output and significantly greater fluid balance, while no significant difference in fluid input was observed.

The median normalized ECVP load was 14.3 mmHg. By using this threshold, we divided the overall population into the high normalized ECVP load group (n = 550, median [IQR]: 16.1 [15.1, 18.0] mmHg) and the low normalized ECVP load group (n = 521, median [IQR]: 13.0 [12.3, 13.7] mmHg). The mortality rate was significantly higher in the high normalized ECVP load group (*p* = 0.003, Fig 4). The incidence of AKI was also significantly higher in the high normalized ECVP load group (92.9% *vs*. 82.3%, *p* < 0.001). There was significant greater 72-hour cumulative fluid intake (4000 [2500, 7000] *vs*. 4000 [2000, 6500] mL, *p* = 0.012), less 72-hour cumulative urine output (3114 [1214, 5461] *vs*. 4363 [2493, 6925] mL, *p* < 0.001) in the high normalized ECVP load group. The 72-hour fluid balance was also significantly greater in the high normalized ECVP load group (1098 [–1418, 4035] *vs*. 46 [–2930, 3462] mL, *p* < 0.001; Fig 5).

**Fig 6:**
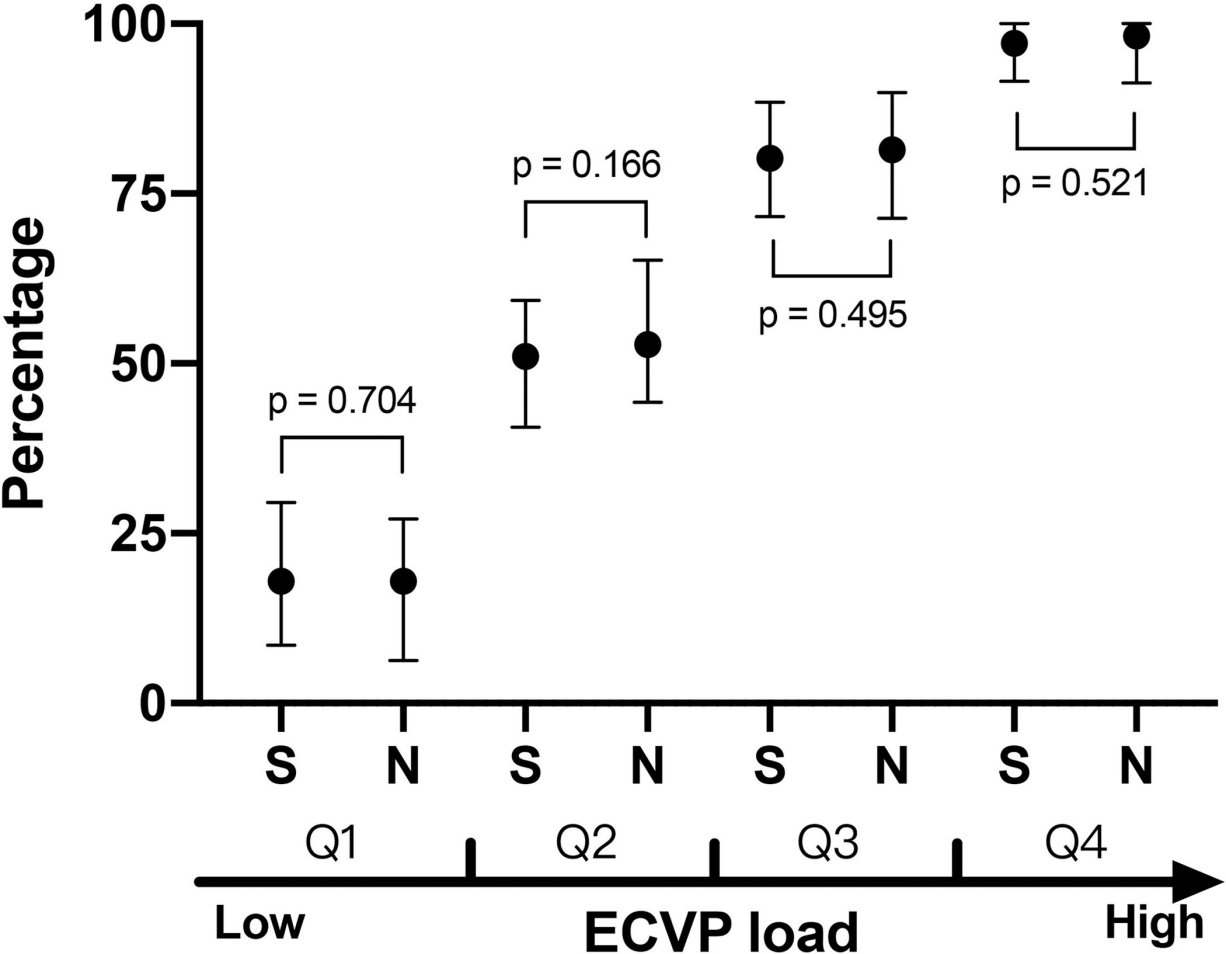
Comparison of the duration of elevated central venous pressure in patients with different elevated central venous pressure load. Patients were divided into four subgroups (Q1 to Q4) according to the quartiles of elevated central venous pressure (ECVP) load. Data were presented as median and interquartile range. The percentage of ECVP duration in total CVP duration is presented on the y-axis and compared between survivors and non-survivors, assuming that patients had comparable ECVP loads within their subgroups. No significant difference was observed in each subgroup. S: survivor, N: non-survivor.

Patients were divided into four subgroups according to the quartiles of ECVP load to compare the duration of ECVP between survivors and non-survivors, with the assumption that patients had comparable ECVP load within their subgroups. There was no significant difference in the duration of ECVP in each subgroup (Q1: 10 [4, 17] *vs*. 8.5 [3, 13.8] hours, *p* = 0.087; Q2: 30.4 [26, 36.5] *vs*. 30 [26.8, 36.2] hours, *p* = 0.729; Q3: 49 [44.6, 53.8] *vs*. 47.1 [44, 52] hours, *p* = 0.062; and Q4: 63 [58.6, 66.6] *vs*. 62.7 [56.6, 66.3] hours, *p* = 0.549). There was also no difference in the percentage of ECVP duration in total CVP duration (Fig 6).

Age, SAPS-II score, minimum hemoglobin concentration, minimum platelet count, maximum international normalized ratio and normalized ECVP load were included in the final multivariate logistic regression model (Table 3). A higher normalized ECVP load is also associated with a higher risk of mortality in the multivariate analysis (odds ratio: 1.058, 95% confidence interval: 1.011 to 1.108; *p* = 0.015).

**Table 3:** Risk factors identified by univariate and multivariate logistic regression.

	Univariate analysis			multivariate analysis		
	OR	95% confidence interval	*p* value	OR	95% confidence interval	*p* value
Age	1.02	(1.012, 1.029)	<0.001	1.02	(1.009, 1.032)	0.001
Female	1.206	(0.935, 1.555)	0.148			
SOFA score	1.095	(1.056, 1.135)	<0.001			
SAPS-II score	1.031	(1.022, 1.041)	<0.001	1.018	(1.007, 1.03)	0.002
Congestive heart failure	1.336	(1.037, 1.722)	0.025			
Chronic pulmonary disease	1.3	(0.995, 1.699)	0.055			
Mild liver disease	1.38	(1.041, 1.829)	0.025			
Severe liver disease	1.753	(1.188, 2.586)	0.005			
Renal disease	1.473	(1.123, 1.933)	0.005			
Diabetes without complication	1.344	(1.022, 1.767)	0.034			
Malignant cancer	2.447	(1.435, 4.173)	0.001			
Maximum lactate	1.082	(1.04, 1.126)	<0.001			
Maximum bilirubin	1.039	(1.008, 1.07)	0.014			
Maximum creatinine	1.088	(1.009, 1.174)	0.029			
Minimum hemoglobin	0.92	(0.864, 0.979)	0.009	0.909	(0.844, 0.98)	0.013
Minimum platelet	0.998	(0.997, 0.999)	0.002	0.998	(0.997, 0.999)	0.006
Maximum international normalized ratio	1.207	(1.097, 1.329)	<0.001	1.168	(1.044, 1.307)	0.007
Minimum mean arterial blood pressure	0.988	(0.98, 0.997)	0.006			
Mean body temperature	0.751	(0.637, 0.886)	0.001			
Minimum pulse O_2_ saturation	0.983	(0.97, 0.996)	0.009			
Normalized ECVP load	1.067	(1.028, 1.106)	0.001	1.058	(1.011, 1.108)	0.015

*SOFA* sequential organ failure assessment, *SAPS-II* simplified acute physiology score-II, *ECVP* elevated central venous pressure

## Discussion

The main findings of the present study were: 1) higher normalized ECVP load was associated with higher mortality; in contrast, the duration of ECVP was not associated with mortality; 2) patients with higher normalized ECVP load had less urine output and more positive fluid balance.

Although numerous studies show the association between higher CVP levels and worse outcomes [6–11], the effect of the duration of ECVP is still unclear. Both the magnitude of ECVP and the duration that ECVP level sustains determine the extent of exposure to ECVP (i.e., the ECVP load). It is natural to assume that both two factors, and their product, might affect the outcomes. In the present study, we investigated the effect of the following factors on mortality: factors indicating the extent of overall CVP/ECVP exposure, which is the product of the CVP value times the duration (i.e., CVP/ECVP load), the duration of such exposure (i.e., CVP/ECVP duration), and the time-weighted average CVP/ECVP level (i.e., normalized CVP/ECVP load). We need the "normalized" parameters to describe the time-weighted average CVP/ECVP because that CVP was not always measured in a fixed-time manner (e.g., once per hour), and a simple calculation of the mean value is insufficient to reflect the actual average CVP exposure. The term “normalized” was therefore adopted from Zhang’s work, which used “normalized lactate load” to describe the time-weighted average lactate level, to describe the process of time-weighted value calculation [17]. In addition, the length of CVP monitoring could be different for each individual because the start time of CVP monitoring may vary based on our study design (theoretically, it can vary between 00:00 to 23:59). This is adjusted by calculating the normalized CVP/ECVP load and the proportion of ECVP duration to CVP duration.

We found no difference in ECVP load between survivors and non-survivors. One possible cause is the difference in the duration of monitoring (but not the exposure), as discussed above. When the ECVP load is divided by the ECVP duration (i.e., "normalized"), it was no longer affected by the difference in the individual CVP duration, and a significant difference in normalized ECVP load was revealed. This finding confirmed that the magnitude of ECVP is strongly associated with worse outcomes in sepsis patients, which has been reported in previous studies [6–11].

On the other hand, no difference was observed in ECVP duration between survivors and non-survivors. Sensitivity analysis was also performed by investigating the difference in ECVP duration in different subgroups, and no difference was found in either lower or higher ECVP load subgroups. These results suggest that the ECVP duration may not associate with mortality in sepsis patients. It is worth discussing why the magnitude of ECVP was associated with mortality while the duration was not. CVP is determined by the complex interplay of the preload, afterload, compliance, and contractility of the right ventricle, venous tone, volume status, abdominal and intrathoracic pressure, and many other factors [18]. For simplicity, it can be interpreted as that CVP is determined by the interaction of the cardiac and venous return functions [19]. Thus, CVP is more likely a "dependent variable" in this interplay rather than an "independent variable." The "independent variables," on the other hand, are intravascular volume and cardiac function. Therefore, CVP provides a useful indicator of how well the heart matches the venous return. A high CVP value (i.e., ECVP) must attribute to either fluid overload or abnormal cardiac function, or both. In other words, extremely high CVP levels indicate the severity of the patient’s cardiac insufficiency. In this regard, it has to be the magnitude rather than the duration of ECVP to determine an unfavorable outcome.

Our finding is consistent with the previous report by Li et al. [7]. In that study only the duration but not the magnitude of ECVP was considered in the sepsis population. In our study, both the magnitude and the duration of ECVP and their product were considered. Our findings expand previous work and provide new data regarding the impact of ECVP on the outcomes of sepsis patients.

The results of three recent multi-center randomized controlled trials (ProCESS, ARISE and ProMISe) have led to the abandonment of the "early goal-directed therapy" (EGDT) strategy [20, 21]. CVP is no longer recommended to guide fluid resuscitation by the most recent SSC guidelines [3]. Instead, dynamic measures of assessing fluid responsiveness are recommended. However, new concerns regarding the concept of fluid responsiveness have been raised: "volume responsive, but does the patient need volume?" [22] What is the significance of CVP in this context? Either way, the fluid infusion must be discontinued because the patient’s heart cannot convert more venous return into cardiac output. In other words, CVP can serve as a safe limit rather than a target of fluid resuscitation [23]. A greater cumulative positive fluid balance was observed in non-survivors in the present study, whereas ECVP is very likely the result of the positive fluid balance. The choice of further (advanced) assessments (e.g., echocardiography, inferior vena cava diameter, cardiac output measurement via thermal dilution, etc.) can be made by monitoring ECVP. Together with further evaluations, the decision whether or not to continue fluid administration or even to achieve a negative fluid balance (i.e., "reverse fluid resuscitation") can be made.

This study has several limitations. First, the study was a retrospective study based on electronic healthcare records and thus limited by the nature of the retrospective design and the source of data used. For this reason, no cause-effect relationships could be established. Second, only patients with an available CVP monitoring till the 72^nd^ hour of ICU stay were included. This is likely to introduce bias because the patients who cannot survive the first 72 hours were excluded. However, our study mainly focuses on the population at high risk of volume overload after initial fluid resuscitation. Third, we used the median to define two separate groups of high or low normalized ECVP load to compare survival. Similarly, quartiles were used to define ECVP load subgroups. Such a grouping method is only for statistical convenience, but there is no physiological basis. Forth, advanced hemodynamic monitoring parameters may be helpful to further evaluate the patient’s cardiac function or volume responsiveness status; unfortunately, these parameters were unavailable in the majority of the patients in this study.

## Conclusions

The magnitude of ECVP is associated with 28-day mortality in patients with sepsis, whereas the duration of ECVP is not. ECVP should be considered as a valuable and easily accessible safety parameter during fluid resuscitation.

## Supporting information

S1 Checklist STROBE 2007 (v4) checklist of items to be included in reports of observational studies in epidemiology*. Checklist for cohort, case-control, and cross-sectional studies (combined).
(DOC)

S1 File Detailed baseline patient characteristics are presented in Table E1. The relationship between normalized central venous pressure load and mortality is presented in Figure E1.
(PDF)

S2 File Stata data file used for the analysis in this study. (DTA)

S3 File Stata.do file used for the calculation in this study. (DO)
