# Supplementary material for: The magnitude, but not the duration of elevated central venous pressure is associated with mortality in sepsis patients: An analysis of the MIMIC-IV database
Source: PLoS One. 2023 Feb 8;18(2):e0281549. doi: 10.1371/journal.pone.0281549 (PMC9907836; doi:10.1371/journal.pone.0281549)
Supplement: S1 File — The relationship between normalized central venous pressure load and mortality is presented in Figure E1. (PDF) [file pone.0281549.s002.pdf]

The magnitude, but not the duration of elevated central venous pressure is associated with mortality in sepsis patients: an analysis of the mimic-iv database

Han CHEN, M.D., Ph.D.; Shu-Rong GONG, M.D.; Xiu-Ling SHANG, M.D.; Ph.D., Jun LI, M.D.; Rong-Guo YU, M.D.*

Supplementary File 1

Table E1 Comparisons of the baseline clinical characteristics between survivors and non-survivors in the first 24 hours

	Survivor (n = 700)	Non-survivor (n = 371)	Overall population (n = 1071)	*p* value
Age (year)	64.4 ± 15.9	69 ± 13.7	66 ± 15.3	< 0.001
Female	283 (40.4)	167 (45)	450 (42)	0.148
Weight (kg)	84.1 ± 24.6	85.7 ± 43.3	84.7 ± 32.3	0.445
Septic shock	578 (82.6)	318 (85.7)	896 (83.7)	0.186
SOFA score	8.7 ± 3.5	9.8 ± 3.5	9.1 ± 3.6	< 0.001
SAPS-II score	49.4 ± 14.4	55.6 ± 13.2	51.6 ± 14.3	< 0.001
**Comorbidities**				
Congestive heart failure	284 (40.6)	177 (47.7)	461 (43)	0.025
Myocardial infarction	133 (19)	97 (26.2)	230 (21.5)	0.007
Peripheral vascular disease	105 (15)	66 (17.8)	171 (16)	0.236
Cerebrovascular disease	72 (10.3)	38 (10.2)	110 (10.3)	0.982

Dementia	20 (2.9)	10 (2.7)	30 (2.8)	0.879
Chronic pulmonary disease	207 (29.6)	131 (35.3)	338 (31.6)	0.055
Rheumatic disease	28 (4)	16 (4.3)	44 (4.1)	0.806
Peptic ulcer disease	29 (4.1)	17 (4.6)	46 (4.3)	0.736
Mild liver disease	167 (23.9)	112 (30.2)	279 (26.1)	0.025
Severe liver disease	62 (8.9)	54 (14.6)	116 (10.8)	0.004
Renal disease	186 (26.6)	129 (34.8)	315 (29.4)	0.005
Diabetes without complication	187 (26.7)	122 (32.9)	309 (28.9)	0.034
Diabetes with complication	63 (9)	37 (10)	100 (9.3)	0.603
Paraplegia	27 (3.9)	6 (1.6)	33 (3.1)	0.044
Malignant cancer	71 (10.1)	56 (15.1)	127 (11.9)	0.017
Metastatic solid tumor	26 (3.7)	32 (8.6)	58 (5.4)	0.001
AIDS	9 (1.3)	2 (0.5)	11 (1)	0.249
**Laboratory results in the first 24 hours**				
Maximum lactate (mmol/L)	3.8 ± 2.9	4.6 ± 3.5	4.1 ± 3.2	< 0.001
Maximum anion gap (mmol/L)	17.8 ± 5.3	19.2 ± 5.4	18.3 ± 5.4	< 0.001
Minimum albumin (g/dL)	2.6 ± 0.7	2.7 ± 0.7	2.6 ± 0.7	0.285
Maximum bilirubin (mg/L)	2.4 ± 3.8	3.3 ± 5.8	2.7 ± 4.7	0.011
Minimum bicarbonate (mmol/L)	18.8 ± 4.9	18.4 ± 5.1	18.6 ± 5	0.249

Maximum bicarbonate (mmol/L)	22.3 ± 4.4	22.3 ± 4.8	22.3 ± 4.5	0.893
Maximum creatinine (mg/dL)	2.1 ± 1.6	2.4 ± 1.7	2.2 ± 1.6	0.028
Minimum glucose (mg/dL)	115 ± 43.5	112.4 ± 48.5	114.1 ± 45.3	0.365
Maximum glucose (mg/dL)	197.8 ± 97	200.6 ± 110.8	198.8 ± 102	0.677
Minimum hemoglobin (g/dL)	9.4 ± 2.1	9.1 ± 2	9.3 ± 2.1	0.008
Minimum platelet (K/uL)	193.6 ± 138.9	167.3 ± 116.9	184.5 ± 132.2	0.002
Maximum potassium (mmol/L)	4.7 ± 0.9	4.8 ± 0.8	4.7 ± 0.8	0.075
Minimum potassium (mmol/L)	3.7 ± 0.6	3.8 ± 0.7	3.8 ± 0.6	0.125
Maximum activated partial thromboplastin time (sec)	55.8 ± 35.4	58.7 ± 36.3	56.8 ± 35.7	0.212
Maximum international normalized ratio	1.9 ± 1.2	2.3 ± 1.7	2.1 ± 1.4	< 0.001
Maximum prothrombin time (sec)	21.1 ± 13.4	24.8 ± 18.4	22.4 ± 15.5	< 0.001
Maximum sodium (mmol/L)	140 ± 6.1	139.3 ± 5.8	139.8 ± 6	0.072
Minimum sodium (mmol/L)	135.6 ± 6	134.9 ± 5.7	135.4 ± 5.9	0.082

Maximum blood urea nitrogen (mg/dL)	40.5 ± 27.8	44.3 ± 26.6	41.8 ± 27.5	0.032
Maximum white blood cell count (K/uL)	18.3 ± 11.7	18.9 ± 14.2	18.5 ± 12.6	0.451
Minimum white blood cell count (K/uL)	12.8 ± 8.7	13.2 ± 10.8	12.9 ± 9.5	0.441
**Vital signs**				
Mean heart rate (bpm)	94.4 ± 17.7	94.8 ± 17.3	94.5 ± 17.5	0.697
Minimum systolic blood pressure (mmHg)	78.6 ± 12.5	75.8 ± 13.5	77.6 ± 12.9	0.001
Minimum diastolic blood pressure (mmHg)	40.6 ± 9.1	38.1 ± 10.7	39.7 ± 9.8	< 0.001
Minimum mean arterial blood pressure (mmHg)	49 ± 14.4	46.4 ± 14.4	48.1 ± 14.4	0.006
Mean respiratory rate (bpm)	21.5 ± 4.5	21.5 ± 4.3	21.5 ± 4.4	0.947
Maximum respiratory rate (bpm)	30.7 ± 7	30.8 ± 6.5	30.7 ± 6.9	0.701
Mean body temperature (°C)	37 ± 0.8	36.8 ± 0.8	36.9 ± 0.8	0.001
Minimum pulse O_2 saturation (%)	90.2 ± 9.3	88.6 ± 9.5	89.6 ± 9.4	0.008

Data are presented as mean ± standard deviation or median (interquartile range) for continuous variables and counts (percentages) for categorical variables.

AIDS acquired immunodeficiency syndrome, *SAPS-II* simplified acute physiology score-II, *SOFA* sequential organ failure assessment

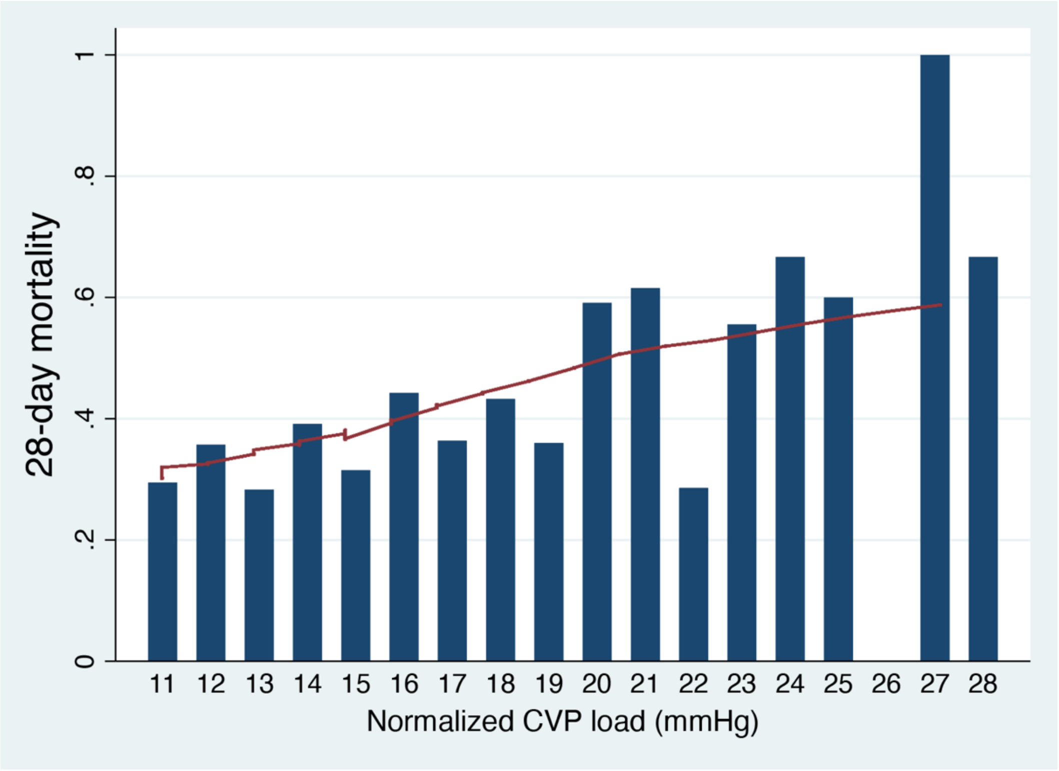

Figure E1 Mortality rate in patients with different normalized central venous pressure load

The bar plot demonstrates the raw mortality rate in each patient subgroup with different normalized central venous pressure (CVP) load. Locally weighted smoothing (Lowess Smoothing) showed a positive correlation between normalized CVP load and mortality.
